# A Multiplex RT-PCR Assay to Detect and Discriminate Porcine Reproductive and Respiratory Syndrome Viruses in Clinical Specimens

**DOI:** 10.3390/v9080205

**Published:** 2017-08-01

**Authors:** Keli Yang, Yongxiang Tian, Danna Zhou, Zhengying Duan, Rui Guo, Zewen Liu, Fangyan Yuan, Wei Liu

**Affiliations:** Key Laboratory of Prevention and Control Agents for Animal Bacteriosis (Ministry of Agriculture), Institute of Animal Husbandry and Veterinary, Hubei Academy of Agricultural Sciences, Wuhan 430064, China; zdn_66@126.com (D.Z.); zy001d@sina.com (Z.D.); hlguorui@163.com (R.G.); liuzwen2004@sina.com (Z.L.); hbxms@126.com (F.Y.); liuwei85@126.com (W.L.)

**Keywords:** porcine reproductive and respiratory syndrome virus, highly pathogenic, vaccine strain, multiplex RT-PCR, viral discrimination

## Abstract

Outbreaks of highly pathogenic porcine reproductive and respiratory syndrome virus (HP-PRRSV) have led to large economic losses in China. The attenuated vaccine (HP-PRRSV JXA1-R) was used to control HP-PRRSV. However, in recent years, co-infection with classical PRRSV (C-PRRSV), HP-PRRSV, and/or HP-PRRSV JXA1-R has been increasing in China, resulting in a significant impact on PRRSV diagnostics and management. To facilitate rapid discrimination of HP-PRRSV JXA1-R from HP-PRRSV and C-PRRSV, a multiplex RT-PCR assay for the visual detection of HP-PRRSV JXA1-R, HP-PRRSV, and C-PRRSV was established and evaluated with reference PRRSV strains and clinical samples. Primer specificities were evaluated with RNA/DNA extracted from 10 viral strains, and our results revealed that the primers had a high specificity for PRRSV. The assay sensitivity was 24 copies/μL for PRRSVs. A total of 516 serum samples were identified, of which 12.21% (63/516) were HP-PRRSV-positive, 2.33% (12/516) were HP-PRRSV JXA1-R-positive, and 1.16% (6/516) were C-PRRSV-positive, respectively, which was completely consistent with the sequencing method. The high specificity, sensitivity, and reliability of the multiplex RT-PCR assay described in this study indicate that it is useful for the rapid and differential diagnosis of HP-PRRSV JXA1-R, HP-PRRSV, and C-PRRSV.

## 1. Introduction

Porcine reproductive and respiratory syndrome (PRRS) is one of the most economically important swine diseases worldwide. The etiological agent of PRRS is the porcine reproductive and respiratory syndrome virus (PRRSV) which belongs to the order *Nidovirales*, family *Arteriviridae* [1]. PRRSV can be divided into European genotype (type 1) and North American genotype (type 2) with Lelystad and VR-2332 as prototypical strains, respectively [2]. The viruses under two genotypes could be further divided into different sub-genotypes according to virus genome characteristics based on phylogenetic analysis [3]. PRRSV was first confirmed in China in 1996 [4], and since then the virus has been found throughout China [5,6]. In May 2006, HP-PRRSV (a highly pathogenic form of PRRSV) severely impacted the pig industry in South China, which led to the death of more than 2 million pigs [7]. Classical PRRSV (C-PRRSV) is the prototypical strain of North American (type 2) genotype VR-2332. The C-PRRSV, HP-PRRSV, and HP-PRRSV JXA1-R strains all belongs to the North American (type 2) genotype, and the PRRSV strains circulating in China are almost all of the North American (type 2) genotype [8,9]. Despite the development and application of modified live vaccines for HP-PRRSV, the virulent HP-PRRSV variants were constantly reported under the massive national immunization campaign [2]. In recent years, co-infection with classical PRRSV (C-PRRSV), HP-PRRSV, and/or HP-PRRSV JXA1-R has been increasing in China, resulting in a significant impact on PRRSV diagnostics and management. Isolation of the pathogenic agents and/or differential serological tests were used for identification and differentiation of PRRSV. However, they are labor-intensive and time-consuming procedures. Molecular typing methods have been developed, and are currently used for rapid detection and identification of PRRSV [10]. Recently, a multiplex real-time RT-PCR based on specific probes was developed for type 1, type 2, and HP-PRRSVs [10]; a SYBR-green-based real-time RT-PCR assay has been developed for detection and differentiation of HP-PRRSV and C-PRRSV [11]; and a one-step RT-PCR assay has been developed for the detection and differentiation of HP-PRRSV and C-PRRSV [12]. However, these assays are not suitable for the differentiation of C-PRRSV, HP-PRRSV, and HP-PRRSV JXA1-R strains. Prompt detection and discrimination of PRRSV in field samples are important for effective PRRS control, thereby reducing the potentially serious economic losses from an outbreak. Therefore, a rapid, convenient, sensitive, and specific diagnostic method to discriminate between C-PRRSV, HP-PRRSV, and HP-PRRSV JXA1-R strains would be extremely useful for the diagnosis and control of PRRSV in China.

In this study, a multiplex RT-PCR assay was developed for the detection and discrimination of C-PRRSV, HP-PRRSV, and HP-PRRSV JXA1-R strains. The proposed method was shown to be a convenient, sensitive, reliable, and suitable tool to aid the prevention and control of PRRS.

## 2. Materials and Methods

### 2.1. Viruses, Cells, and Reagents

C-PRRSV strain CH-1a (GenBank ID: AY032626) is the first wild-type strain isolated from China, and was kindly provided by Hanzhong Wang (Wuhan Institute of Virology, Chinese Academy of Sciences, Wuhan, China). PRRSV strain 07HBEZ is a highly pathogenic North American-type PRRSV, and was isolated in 2007 (GenBank ID: FJ495082.2). HP-PRRSV JXA1-R strain was isolated from highly pathogenic porcine reproductive and respiratory syndrome vaccine, live (JXA1-R) (Pulike Biological Engineering Inc., Luoyang, China).

Marc-145 cells were cultured and maintained in DMEM supplemented with 10% newborn calf serum (Gibco) at 37 °C in a humidified 5% CO_2_ incubator. Other viruses, including classical swine fever virus (CSFV), pseudorabies virus (PRV), porcine circovirus type 2 (PCV2), porcine parvovirus (PPV), Japanese encephalitis virus (JEV), rotavirus (RV), porcine epidemic diarrhea virus (PEDV), stored in our laboratory, were used to confirm the specificity of the proposed multiplex RT-PCR assay.

### 2.2. Clinical Specimen Collection

A total of 516 serum samples were obtained from 10 pig farms in Hubei Province, central China, from June to August 2016. All samples used in this study were collected in accordance with international standards for animal welfare.

The pigs were immunized with live vaccine of HP-PRRSV JXA1-R strain (Pulike Biological Engineering Inc.) according to the manufacturer’s instructions at about 30 days old. The serum samples were collected from the pigs when they were about 50 days old. Before samples were collected, there were no clinical signs of PRRSV in the farms.

### 2.3. Primers Design

The primers were designed using Primer Premier 5.0 (Primer Biosoft International, Palo Alto, Santa Clara, CA, USA) based on the PRRSV sequences in GenBank database to amplify the discontinuous 30-aa deletion in the *NSP2* gene between HP-PRRSV and C-PRRSV genome sequences, and the nucleotide fragments (13,380–13,900nt) of HP-PRRSV and HP-PRRSV JXA1-R strain, respectively (Table 1). The specificity of the primers was confirmed against random nucleotide sequences obtained by a BLAST search in the GenBank database. The PCR-amplified HP-PRRSV and C-PRRSV products were 229 and 319 bp, respectively, and the PCR-amplified products of HP-PRRSV JXA1-R strain were two bands of 229 bp and 620 bp.The primers were synthesized by Sangon Biotech Co., Ltd. (Shanghai, China).

### 2.4. Nucleic Acid Extractions

Viral RNA samples for multiplex RT-PCR were extracted from the serum samples using the TaKaRa MiniBEST Viral RNA Extraction Kit Ver.4.0 (TaKaRa, Dalian, China) according to the manufacturer’s protocol. Total RNA was eluted with 40 μL of diethylpyrocarbonate-treated water and stored at −80 °C until use.

Viral genomic RNA or DNA to test the specificity of the multiplex RT-PCR assay were extracted from cell cultures infected by each virus using the TaKaRa MiniBEST Viral RNA/DNA Extraction Kit Ver. 4.0 (TaKaRa, Dalian, China) according to the manufacturer’s instructions.

### 2.5. First Strand cDNA Synthesis

First strand cDNA was synthesized from 5 μg total RNA using PrimeScript™ 1st Strand cDNA Synthesis Kit (TaKaRa) following the manufacturer’s protocol with OligodT primer.

### 2.6. Single PCR and Plasmid Template Construction

The PCR reaction was conducted in a 25 µL mixture including 2.5 µL 10× PCR buffer, 2 µL 10 mM of each dNTPs, 0.5 µL of each 10 µM primer (Table 1), 1.25 U of Taq DNA polymerase (5 U/µL) (TaKaRa), 2.5 µL of the cDNA, and distilled water was added to 25 µL.

The amplifications were performed in a thermal cycler (Bio-Rad, Hercules, CA, USA) under the following conditions: after initial denaturation at 95 °C for 5 min, 35 cycles were conducted at 95 °C for 30 s, 58 °C for 45 s, and 72 °C for 45 s, followed by a final extension at 72 °C for 10 min. The PCR products were detected by electrophoresing through 1.0% agarose gel in 1× TAE (40 mM Tris-aceate, 1 mM EDTA, pH8.0). Each specific viral target fragment was cloned into the plasmid pMD18-T (TaKaRa). The constructed recombinant plasmids were sequenced and confirmed to use as standard templates for optimization of the following PCR assays.

### 2.7. Optimization of the MultiplexRT-PCR Assay

Based on the established single PCR, the multiplex RT-PCR was optimized by varying single parameters while other parameters were maintained. The evaluated parameters and ranges in concentrations included: primers, 2–30 pM; dNTPs, 100–400 μM, and TaKaRa*Taq* DNA polymerase, 0.5–2.5 U. The effects of annealing temperature (range: 55–62 °C) and number of cycles (range: 25–35 cycles) were also determined experimentally. The PCR products were detected by electrophoresing as described above. Negative control using distilled water instead of template cDNA was run with the test. 

### 2.8. Specificity of the Proposed Multiplex RT-PCR Assay

Specificity of the multiplex RT-PCR assay was determined by analyzing 10 different viral strains and ddH_2_O as negative control. C-PRRSV, HP-PRRSV, and HP-PRRSV JXA1-R strains were identified by sequencing, and the other virus strains (CSFV, PRV, PCV2, PPV, JEV, RV, and PEDV) were verified by serological or PCR methods. Viral RNA extracted from C-PRRSV-, HP-PRRSV-, and HP-PRRSV JXA1-R infected cell supernatants with approximate viral titers of 10^3^ TCID_50_/mL were analyzed with the multiplex RT-PCR assay as described above. 

### 2.9. Sensitivity of the Proposed Multiplex RT-PCR Assay

To assess the sensitivity of the proposed assay, the C-PRRSV, HP-PRRSV, and HP-PRRSV JXA1-R strains were determined in Marc-145 cells grown in 96-well plates, and the TCID_50_ was calculated using the method of Reed and Muench [13]. 

Total RNA from the virus samples was extracted and analyzed with the sensitivity of the proposed multiplex RT-PCR assay. The RNA concentration was determined by spectrophotometry, and the exact number of RNA molecules was calculated. Then 10-fold serial dilution was performed from 2.4 × 10^5^ copies/μL to 2.4 × 10^−1^ copies/μL and used as templates for multiplex RT-PCR. The lowest amount of RNA detectable under the conditions described above was defined as the sensitivity of the multiplex RT-PCR assay.

### 2.10. Detection of PRRSV in Clinical Specimens by the Multiplex PCR

To evaluate the feasibility of multiplex RT-PCR detection of PRRSV, 516 clinical specimens described above were analyzed using the multiplex RT-PCR assay. To verify the accuracy of the developed protocol, all of the RT-PCR products from positive samples were cloned into the plasmid pMD18-T and sequenced by Sangon Biotech Co., Ltd (Shanghai, China). All of the negative samples were further detected using the one-step RT-PCR in our previous study [12].

## 3. Results

### 3.1. MultiplexRT-PCR Assay Conditions

The optimal parameters of multiplex RT-PCR assay were investigated. A final 25-µL volume of the multiplex RT-PCR master mix contained of 2.5 μL of 10× buffer, 0.25 μL of *Taq* polymerase (5 U/μL), 2.5 μL of cDNA template, 2μL of dNTPs (10 mM), 0.5μL of forward and reverse primers (10 μM), and 16.75 µL nuclease-free water in each reaction tube. Master mixes were maintained on ice at all times prior to PCR analysis. An optimized experimental protocol on a Peltier Thermal Cycler machine (Bio-Rad, Hercules, CA, USA) consisted of a denaturation program (95 °C for 5 min) and an amplification program repeated 35 times (denaturation at 95 °C for 30 s, 58 °C for 40 s, and elongation at 72°C for 45 s), followed by a 10-min extension at 72°C. 

**Figure 1:**
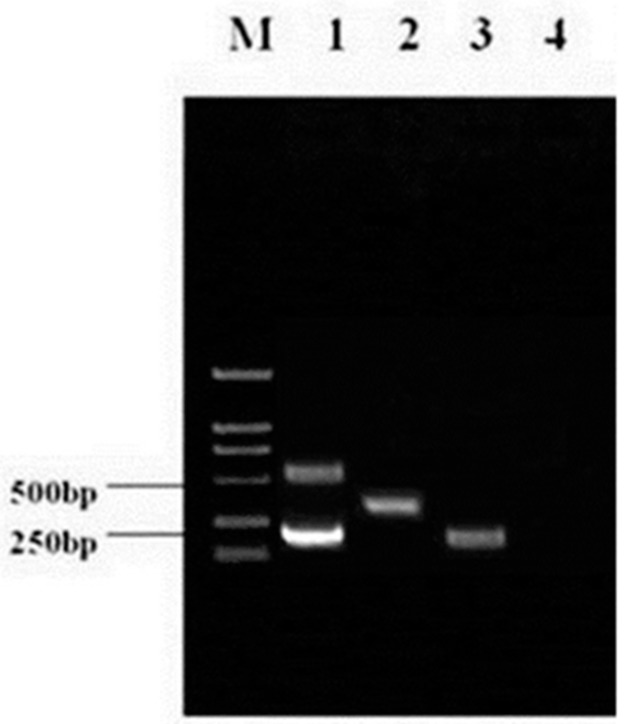
The results of multiplex RT-PCR in optimization conditions.Lane M, DL2000 DNA marker; lane 1, highly pathogenic porcine reproductive and respiratory syndrome virus (HP-PRRSV) strain JXA1-R; lane 2, classical PRRSV (C-PRRSV) strain CH-1a; lane 3, HP-PRRSV strain 07HBEZ; lane 4, ddH_2_O.

HP-PRRSV JXA1-R cDNA, HP-PRRSV cDNA, C-PRRSV cDNA, and a negative control reaction mixture without template were analyzed using the above protocol, and the PCR amplification results are illustrated in Figure 1.

### 3.2. Specificity of the Proposed Multiplex RT-PCR Assay

**Figure 2:**
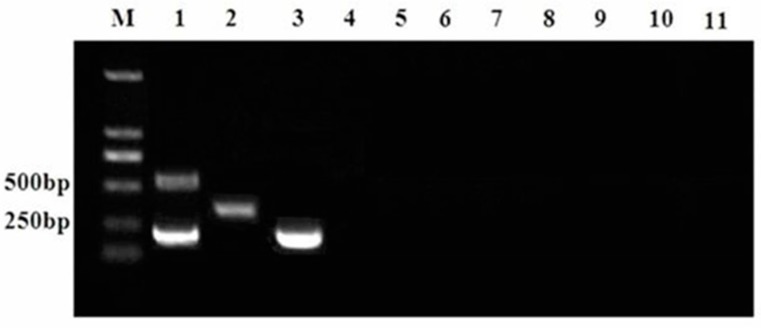
Specificity of multiplex RT-PCR. Lane M, DL2000 DNA marker; lane 1, HP-PRRSV JXA1-R; lane 2, C-PRRSV strain CH-1a; lane 3, HP-PRRSV strain 07HBEZ; lane 4, CSFV; lane 5, PRV; lane 6, PCV2; lane 7, PPV; lane 8, JEV; lane 9, RV; lane 10, PEDV; lane 11, ddH_2_O.

The specificity of the primer pairs for each virus was analyzed using the proposed multiplex RT-PCR assay. As illustrated in Figure 2, the multiplex RT-PCR assay was specific for the target virus because no amplification occurred with CSFV, PRV, PCV2, PPV, JEV, RV, PEDV, or ddH_2_O (lanes 4–11), whereas the HP-PRRSV JXA1-R, C-PRRSV, and HP-PRRSV target genes were specifically amplified using the defined primer pairs (lanes 1–3).

### 3.3. Sensitivity of Multiplex RT-PCR

The sensitivity of the multiplex RT-PCR assay was defined as the minimum detectable RNA molecules concentration at which a positive amplification product could be detected. 

**Figure 3:**
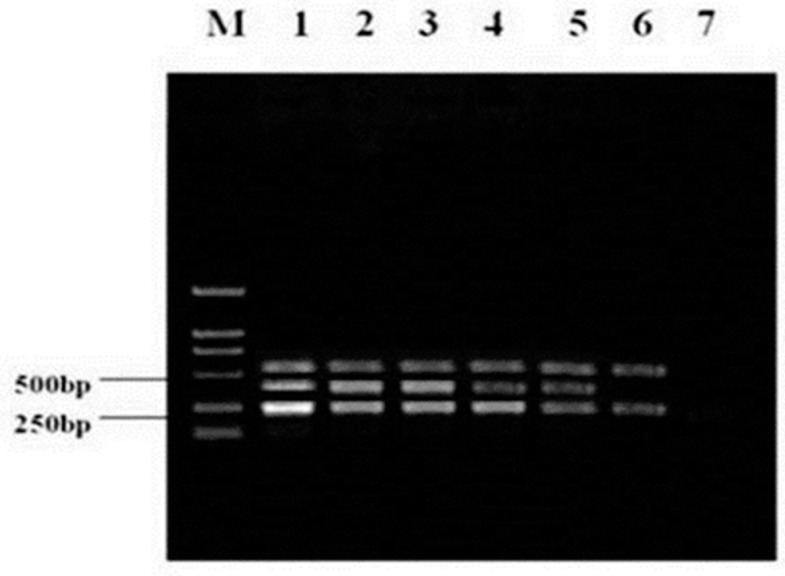
Sensitivity of multiplex RT-PCR for PRRSV Lane M, DL2000 DNA marker; lanes 1–7 are: 1, 2.4 × 10^5^; 2, 2.4 × 10^4^; 3, 2.4 × 10^3^; 4, 2.4 × 10^2^; 5, 2.4 × 10^1^; 6, 2.4 × 10^0^; 7, 2.4 × 10^−1^ copies/μL.

Upon 10-fold serial dilution, RNA standards with known copy numbers (2.4 × 10^5^ copies/μL to 2.4 × 10^−1^ copies/μL) were synthesized first strand cDNA and used for multiplex RT-PCR. As shown in Figure 3, multiplex RT-PCR successfully detected as little as 24 copies/μL of RNA molecules, determined by the agarose gelelectrophoresis for the HP-PRRSV JXA1-R, C-PRRSV, and HP-PRRSV. The results demonstrated that the sensitivity of the multiplex RT-PCR was 24 copies/μL for HP-PRRSV JXA1-R, C-PRRSV, and HP-PRRSV.

### 3.4. Detection of Viruses in Clinical Specimens

A total of 516 clinical specimens were tested by the multiplex RT-PCR assay with optimal parameters. The results were as follows: HP-PRRSV RNA was detected in 63 (12.21%) of 516 serum samples, HP-PRRSV JXA1-R RNA was detected in 12 (2.33%) of 516 serum samples, and C-PRRSV RNA was detected in 6 (1.16%) of 516 serum samples, respectively, which were 100% correlated with the sequencing method (Table 2). The results of one-step RT-PCR for negative samples were same as those of the multiplex RT-PCR assay we developed. The results obtained by the multiplex RT-PCR method and subsequent sequencing further indicated the accuracy of the developed method.

Additionally, two HP-PRRSV JXA1-R and HP-PRRSV positive, one HP-PRRSV and C-PRRSV positive, and two HP-PRRSV JXA1-R and C-PRRSV positive clinical samples were detected in samples from four pig farms (Table 2). The rate of co-infection was 0.97% (5/516) in all of the detected clinical specimens.

## 4. Discussion

PRRSV has obsessed the pig industry for decades and leads to massive economic losses all over the world. In 2006, a highly-pathogenic PRRSV occurred in China, affected over 2,000,000 pigs with about 400,000 fatal cases [7]. The outbreaks observed were further characterized by a rapid spread within the affected provinces, and it was observed that pigs of all ages were affected [14]. Due to its high rates of mutation and recombination events, the protection ability of PRRSV vaccines was compromised if infection froma heterologous virus occurred [15]. In order to prevent PRRS, six live attenuated PRRSV vaccines including PRRSMLV, CH-1R, and R98 for C-PRRSV, and JXA1-R, HuN4-F112, and TJM-F92 specific for HP-PRRSV are currently marketed in China [16], and the JXA1-R strain has been widely used in recent years. However, co-infection with classical PRRSV (C-PRRSV), HP-PRRSV, and/or HP-PRRSV JXA1-R has been increasing in China, resulting in a significant impact on PRRSV diagnostics and management.

PRRS diagnosis is typically accomplished by viral isolation followed by immunochromatochemical analysis, serological methods, and/or conventional RT-PCR. However, viral isolation is complex, labor intensive, and time-consuming. Immunochromatochemical and serological methods have low specificity and/or sensitivity [17]. To overcome these shortcomings and obtain more accurate diagnoses, real-time RT-PCR assays specific for the highly-pathogenic PRRSV have been developed [18,19,20], and some qRT-PCR assays and one-step RT-PCR assays have been developed for the detection and differentiation of HP-PRRSV and C-PRRSV [10,11,12]. These tests are highly specific and sensitive, but cannot distinguish C-PRRSV, HP-PRRSV, and HP-PRRSV JXA1-R strains coexisting in Chinese swine herds. 

In the present study, we developed an efficient and sensitive multiplex RT-PCR assay for the detection and discrimination of C-PRRSV, HP-PRRSV, and HP-PRRSV JXA1-R strains using two specific primer pairs—one of which was designed in our previous study [12]. The PCR products produced from the specific primers are distinct for each PRRSV strain, and no amplification occurred with non-target viruses or ddH_2_O, which can be visualized and easily differentiated by agarose gel electrophoresis checking. In addition, all the specific PCR products obtained by analyzing the clinical specimens were also cloned into pMD18-T and sequenced to further confirm the specificity of the multiplex assay. Generally, the presence of more than one pair of primers in the same reaction mix may limit the sensitivity or cause preferential amplification of specific targets [21], and the sensitivity of the multiplex PCR is usually approximately 10-fold lower than that of a single PCR [22]. Fortunately, the established multiplex RT-PCR assay in the study was as sensitive as the single PCR in our previous study [12] with a detection limit of 24 copies/µL for PRRSV, which suggested a desired primer design and proper optimization of the multiplex RT-PCR assay developed. Moreover, the sensitivity was similar to that of a real-time RT-PCR-based assay for the same virus (the sensitivity of which was 20 copies/μL) [10], and was higher than a multiplex PCR (the sensitivity of which was 800 copies/μL) [23] and a RT-qPCR assay (the sensitivity of which was 100 copies/μL) [24]. As we know, there are many factors that can affect the sensitivity of PCR assays, such as the nucleic acid extraction method, the cycles of PCR, and the PCR products detection method, etc. The sensitivity difference between the multiplex PCR we developed and the RT-qPCR assay [24] maybe caused by the factors above, and the sensitivity difference between the multiplex PCR we developed and another multiplex PCR assay [23] maybe mainly caused by the nucleic acid extraction method. In the present study, when assessing diagnostic performances, the sensitivity of the proposed multiplex RT-PCR assay was 24 copies/μL; that is to say, the samples with the virus below 24 copies/μL could not be detected as positive. Additionally, the sensitivity of the multiplex RT-PCR in this study, is similar with that of the one-step RT-PCR in our previous study [12], which was 25 copies/μL for both HP-PRRSV and C-PRRSV. The difference between the two assays is that the one step RT-PCR assay can detect and discriminate HP-PRRSV and C-PRRSV, while the multiplex RT-PCR assay can detect and discriminate not only HP-PRRSV and C-PRRSV, but also HP-PRRSV JXA1-R. However, the PRRSV has genetic variability; when a new variant strain of PRRSV appears, it is possible that the multiplex RT-PCR would be unable to detect and discriminate it. This facet will also need further research.

In the present study, 516 specimens were detected using the multiplex RT-PCR, and the results were 100% consistent with that of the sequencing method. In our clinical specimens, 12.21% (63/516) were HP-PRRSV-positive, 2.33% (12/516) were HP-PRRSV JXA1-R-positive, and 1.16% (6/516) were C-PRRSV-positive, respectively, which was completely consistent with that of the sequencing method. The results also indicated that the PRRSV infection was still severe in Hubei Province, Central China.

At present, several commercial PRRSV vaccines are used in pig farms in China, including modified live vaccines (MLV) and inactivated vaccines (IV). The use of vaccines in piglets has been more controversial, and is limited by the dynamics of viral circulation in the farm. When the flow of viremic piglets from maternities to nurseries is high, most piglets will be infected before the establishment of an active immunity due to vaccination [25]. Some studies have dealt with the evaluation of virus transmission to vaccinated and unvaccinated pigs [25,26,27,28]. Thus, in controlling PRRS, it is important to develop an assay to discriminate different PRRSV strains in clinical specimens.

Currently, the epidemic of atypical PRRS has not been completely controlled in China [14]. The highly pathogenic PRRSV may continue to impact the Chinese swine industry for an extended period [12]. PRRS is one of the most important pig diseases worldwide. The causative PRRSV is rapidly evolving and there is an urgent need for the development of quicker and more efficacious assay to discriminate PRRSV different strains to improve PRRS control.

Control of PRRSV infection in pig farms mainly relies on four pillars:biosecurity, diagnosis, herd management and immunization. Of these, diagnosisis essential to detect the pathogen and to limit the impact of the infection within an infected herd. Thus, we developed the proposed multiplex RT-PCR to aid in PRRS prevention and control. In this study, since we could discriminate PRRSV different strains in the clinical specimens, we believed that the multiplex RT-PCR could be used for the detection of PRRSV, and useful in implementing methods to prevent the transmission of the disease.

In summary, the proposed multiplex RT-PCR is a convenient, rapid, efficient, sensitive, and highly specific assay for the identification of different PRRSV strains. The size of amplified product is sufficient to distinguish the different PRRSV strains. Our method showed great promise not only in laboratory testing, but also in field and clinical applications. The simplicity and accuracy of the test make it a powerful tool for PRRSV detection and control in China.

## Figures and Tables

**Table 1:** The primer sequences for multiplex RT-PCR.

Primer	Primer Sequences (5′–3′)	Origin/Target Gene	Location	Products	Source
Nsp2-F	TGAYGGGCGACAATGTCC	PRRSV CH-1a (GenBank:AY032626)/Nsp2	2745–2762(AY032626)	319 bp (AY032626)	Previous study [12]
			1406–1423(FJ495082.2)		
Nsp2-R	CGCAGACAAATCCAGAVG	PRRSV 07HBEZ (GenBank:FJ495082.2)/Nsp2	3064–3047(AY032626)	229 bp (FJ495082.2)	
			1635–1618(FJ495082.2)		
JXA1-F	ATTTGAATGTTCGCACGGTCTC	PRRSV 07HBEZ (GenBank:FJ495082.2)/GP4	13,380–13,401	None (FJ495082.2)	This study
JXA1-R	CCGCTGAAACTCTGGTTAAAGG	PRRSV JXA1-P170 (GenBank:JQ804986.1)/GP5	13,879–13,900	620 bp (JQ804986.1)	

**Table 2:** Detection rates of clinical specimens by multiplex RT-PCR and sequencing method.

		Multiplex RT-PCR			Sequencing Method			
Pig Farm	No. of Specimens	HP-PRRSV JXA1-R Positive (%)	HP-PRRSV Positive (%)	C-PRRSV Positive (%)	HP-PRRSV JXA1-R Positive (%)	HP-PRRSV Positive (%)	C-PRRSV Positive (%)	Concordance Rate (%)
1	56	1 (1.79)	6 (10.71)	0 (0)	1 (1.79)	6 (10.71)	0 (0)	100
2	60	2 ^a^ (3.33)	11 ^a^ (18.33)	2 (3.33)	2 ^a^ (3.33)	11 ^a^ (18.33)	2 (3.33)	100
3	45	1 (2.22)	5 (11.11)	0 (0)	1 (2.22)	5 (11.11)	0 (0)	100
4	58	2 ^a^ (3.45)	9 ^a^ (15.52)	1 (1.72)	2 ^a^ (3.45)	9 ^a^ (15.52)	1 (1.72)	100
5	32	0 (0)	3 (9.38)	0 (0)	0 (0)	3 (9.38)	0 (0)	100
6	35	0 (0)	5 (14.29)	0 (0)	0 (0)	5 (14.29)	0 (0)	100
7	49	1 (2.04)	2 ^b^ (4.08)	1 ^b^ (2.04)	1 (2.04)	2 ^b^ (4.08)	1 ^b^ (2.04)	100
8	57	2 (3.51)	6 (10.53)	0 (0)	2 (3.51)	6 (10.53)	0 (0)	100
9	66	2 ^c^ (3.03)	10 (15.15)	2 ^c^ (3.03)	2^c^ (3.03)	10 (15.15)	2 ^c^ (3.03)	100
10	58	1 (1.72)	6 (10.34)	0 (0)	1 (1.72)	6 (10.34)	0 (0)	100
Total	516	12 (2.33)	63 (12.21)	6 (1.16)	12 (2.33)	63 (12.21)	6 (1.16)	100

^a^ One sample in the farm was HP-PRRSV JXA1-R and HP-PRRSV positive, the positive ratio was 1.67% (1/60) and 1.72% (1/58), respectively; ^b^ One sample in the farm was HP-PRRSV and C-PRRSV positive, the positive ratio was 2.04% (1/49); ^c^ Two samples in the farm were HP-PRRSV JXA1-R and C-PRRSV positive, the positive ratio was 3.03% (2/66).

## Abbreviations

PRRSV	porcine reproductive and respiratory syndrome virus
CSFV	classical swine fever virus
PRV	pseudorabies virus
PCV2	porcine circovirus type 2
PPV	porcine parvovirus
JEV	Japanese encephalitis virus
RV	rotavirus
PEDV	porcine epidemic diarrhea virus
